# When Appropriate Statistical Analysis is Dismissed

**DOI:** 10.5812/hepatmon.7024

**Published:** 2012-08-12

**Authors:** Mohamad Amin Pourhoseingholi, Ahmad Reza Baghestani, Mohsen Vahedi

**Affiliations:** 1 Gastroenterology and Liver Diseases Research Center, Shahid Beheshti University of Medical Sciences, Tehran, IR Iran; 2 Department of Mathematic, Islamic Azad University - South Tehran Branch, Tehran, IR Iran; 3 Department of Epidemiology and Biostatistics, School of Public Health, Tehran University of Medical Sciences, Tehran, IR Iran

**Keywords:** Sample Size, Chi-Square Distribution

**Dear Editor,**

We read with interest the paper entitled “Prevalence of Hepatitis D Virus Infection among Hepatitis B Virus Infected Patients in Qom Province, Center of Iran” by Ghadir et al. (1) which was performed to determine the prevalence of hepatitis D in the general population of Qom province and the potential risk factors for acquiring HDV. They found 48 subjects (1.3%) suffered from hepatitis B and only one HBsAg-positive case with HDV infection (0.03%) and concluded that the prevalence of hepatitis D in Qom is the lowest in Iran. Despite this fact that the only person who was positive for hepatitis D was a 31-years-old woman, living in Qom but originated from Afghanistan (opposite of study’s method which claimed that those who were not Iranian were excluded from the study) (1), there are some skeptical concerns regarding the comparison with hepatitis B positive and hepatitis D positive for potential risk factors. The authors compared one person (as hepatitis D group) to 49 persons (as hepatitis B group) for risk factors using chi-square and Fisher exact test and they reported one significant association between Hepatitis D and Tattoo. The results of this study are violated by the small sample size. If the sample size is too small, a well conducted study may fail to answer its research hypothesis or may fail to detect important effects and associations (2). Besides, for using Chi Square test, the total number in the study should exceed 30 cases, and each cell should have at least five cases (3). In the absence of these numbers, the assumptions of Chi Squares distribution cannot be assured and there is a possibility of misinterpretation, and power decrase of the study. However if the sample size for a contingency table is big enough for a 2 × 2 independent test; the chi-square analysis gives P-values that are considerably lower than that of the other exact tests. We do not know which analysis was performed on the data in this paper (Chi Squares or Fisher exact test), however repetition of the analysis for relation between hepatitis and tattoo indicated that the authors reported the P-value of Likelihood Ratio, instead of Fisher exact test (which was not significant according to Fisher). Therefore the result was totally skeptical. One solution is to use Yates’ correction for continuity, which is sometimes just known as the continuity correction. It works quite well, yielding P-values that are quite close to those of the exact binomial test and the another alternative is Fisher exact test, however when sample size is too small, the results could not reflect the real correlation.

Finally we recommend that while there are user-friendly statistical packages, investigators should consult biostatistician at the time of data analysis to avoid the errors due to small sample size or inappropriate statistical tests.
